# 

**Published:** 1918

**Authors:** 


### TABLE OF CONTENTS

Surgical Dyspepsia——A Case Report..........349
Guarding The Portals of Entry..........353
Quarantinable Diseases..........361
Treatment of Compound Fractures..........368
Acute Nephritis Complicating Infectious Diseases 375

